# Long-term Outcomes of Sleeve Gastrectomy in Adolescent Patients: The Effect of Weight Loss in Younger Years to Outcomes in Adulthood

**DOI:** 10.1186/s12893-023-02006-6

**Published:** 2023-04-28

**Authors:** Salman Al Sabah, Eliana Al Haddad, Sameera Shuaibi, Iman Qadhi, Lulwah Al-Saidan, Ali Khayat

**Affiliations:** 1 Department of Surgery, Kuwait University, Kuwait City, Kuwait; 2 Al Amiri Hospital, Kuwait City, Kuwait

**Keywords:** Sleeve gastrectomy, Adolescent, Childhood obesity, Bariatric surgery.

## Abstract

**Background:**

Childhood obesity is associated with a variety of complications that see their light throughout adulthood. Due to the serious side effects of these morbidities, early intervention is essential. Laparoscopic sleeve gastrectomy (SG) is a safe and effective procedure for the treatment of obesity, however, the long-term data on its use in adolescents is lacking in the literature.

**Methods:**

A retrospective analysis was conducted on all patients that underwent SG aged between 12 and 21 years old at a public hospital in Kuwait. Data on their weight and comorbidities was collected and analyzed.

**Results:**

164 adolescent patients with a mean age of 19 underwent SG. 71% of the patients were female, while the mean weight at surgery was 128.6 kg, corresponding to a BMI of 47.8 Kg/m2. 32% of patients had a starting BMI more than 50, while 6.7% had a BMI over 60. The highest weight loss was achieved at 18 months post-op, corresponding to an EWL of 82.66%. On long-term follow-up, weight loss was maintained over the 13 years post-op. Obstructive sleep apnea resolved in 75% of the patients while hypertension persisted in the 2 patients who were diagnosed with it pre-op. 21 patients developed gastro-esophageal reflux disease 5.7 years post-op, while 20 patients were treated for gall bladder stones 4.4 years post-op.

**Conclusion:**

It is of ample importance to tackle obesity during childhood before complications ensue later in life. Bariatric surgery, specifically SG, has been found to be an effective and safe weight loss tool, with sustained long-term weight maintenance and resolution of early comorbidities.

## Key Points

Laparoscopic sleeve gastrectomy is an effective procedure for the long-term treatment of obesity.

Childhood obesity carries its effects into adulthood, and can have detrimental effects throughout the life of a patient.

SG shows effective long-term maintained weight loss when performed in adolescent patients.

## Introduction

Obesity has become one of the leading causes of morbidity and mortality amongst all age groups in the world. Furthermore, the prevalence of obesity has doubled worldwide since 1980 [1, 2]. Considering obesity in the younger population, it has been established that obesity in adolescents is more common among higher income countries and in adults who have been diagnosed with obesity in their childhood. Obese children have an approximate 77% increased risk of becoming obese adults in comparison to 7% of children who are not obese [3]. From an economical perspective, obese adolescents add to governmental financial burdens around the world, with an estimated increased health cost of 14 billion dollars a year [3, 4].

Childhood obesity is associated with a variety of complications that manifest later in adulthood such as hypertension, diabetes, obstructive sleep apnea, in addition to poor self-esteem and discrimination [5]. Due to the serious side effects of these morbidities, lifestyle modifications including diet and exercise are essential. However, failure of these life style modifications in some patients may lead to seeking surgical intervention as early as possible to avoid consequential life-long health problems. Data from the American Society for Metabolic and Bariatric Surgeons (ASMBS) was able to show that 53% of bariatric surgeons had performed bariatric surgery on obese adolescents in 2005 alone [5]. While there has been a substantial increase in demand for bariatric surgeries worldwide, only extremely obese adolescents are presently elected for surgical interference according to current guidelines of the International Pediatric Endo-surgery Group (IPEG) [6].

Amongst the surgeries performed on adolescent patients, laparoscopic sleeve gastrectomy (SG) is more regularly performed due to its effectivity and relative simplicity [7]. In addition, data regarding Roux-en-Y gastric bypass and gastric banding is considerably available in the adolescent population [8].

In contrast, due to limited literature on laparoscopic sleeve gastrectomy amongst adolescents, we aimed to study the long-term trends in weight loss and resolution of comorbidities 10 years post SG within the adolescent population in order to gain a better insight on the overall efficacy of this practice.

## Methods

A retrospective analysis was conducted on all patients that underwent SG between the dates of October 2008 and October 2021 aged between 12 and 21 years old at a public hospital in Kuwait. Ethical approval to conduct the study was obtained from the Ministry of Health and Kuwait Institute for Medical Specialization Ethical Approval Board.

### Inclusion criteria

Patients were examined by a multidisciplinary team to assess their eligibility for undergoing an SG. Childhood and adolescent obesity was defined according to growth charts provided by the Centre for Disease Control (CDC) [9], as well as body mass index. Obesity was defined as having a BMI equal to or greater than the 95th percentile for age and gender. Class III (severe obesity) was defined as > 140% of the 95th percentile for age and gender. Class II obesity was defined as > 120% of the 95th percentile, and Class I obesity was defined as > 95th percentile Parameters that made patients eligible included (1) a body mass index (BMI) of ≥ 140% percentile for age and gender or ≥ 120% percentile with associated comorbidities, (2) failure to achieve clinically significant weight loss after at least 6 months of an attempted weight management regimen/s (3) a positive psychological examination.

### Pre-operative evaluation

A comprehensive laboratory workout was performed on all patients pre-operatively and included a complete blood count, renal and liver profiles, thyroid function tests, coagulation profile, and upper endoscopy to evaluate for GERD, hiatal hernia and pathologic examinations, along with Helicobacter pylori CLO test. These procedures were followed for all patients prior to undergoing SG.

### Definition of co-morbidities – diabetes

Diabetes was defined as: (1) Impaired glycaemia or impaired glucose tolerance (2) Insulin treatment (3) oral antidiabetics (OAD) & insulin treatment or (4) Oral hypoglycaemics.

### Definition of co-morbidities – GERD

Gastro-esoophageal reflux disease was defined as patients that either where on: (1) Daily medication (H2 receptor antagonists (H2RA) or proton pump inhibitors (PPI)) (2) Intermittent medication. Or (3) Intermittent symptoms; no medication. All patients undergoing a sleeve gastrectomy at our institution are evaluated for reflux pre-operatively. If the reflux is Grade A or lower, we would still perform a sleeve gastrectomy on the patients given that the likely etiology is due to obesity itself creating pressure on the abdomen. If grade B or lower reflux is detected, we perform further evaluation for the reflux and place the patients on a PPI regimen as previously described [10]. The patients are then re-evaluated for reflux and if it is resolved, we go ahead with the sleeve gastrectomy planned, however, if the reflux is persistent, then a sleeve gastrectomy is not recommended, and the patients are offered a Roux-en-Y gastric bypass instead.

### Definition of co-morbidities – hypertension (HTN)

Hypertension was defined as either: (1) Treated hypertension (2) Untreated hypertension.

### Definition of co-morbidities – obstructive sleep apnea (OSA)

Obstructive Sleep Apnea was defined according to in-home sleep apnea monitoring devices. Sleep assessments were performed for one night using the Nox T3 device. The device is a type 3 sleep monitor measuring airflow via a nasal cannula; respiratory effort via chest and abdominal belts; body position and activity via an integrated accelerometer; and pulse and oxygen saturation via an oximeter. All sleep examinations were analyzed manually by the same registered polysomnographic technologist (RPSGT) according to the pediatric respiratory rules defined by the American Academy of Sleep Medicine [11]. The RPSGT was blinded to the BMI of the children. Apneas were identified if there was a ≥ 90% drop in airflow for the duration of at least two breaths. Obstructive apneas were defined as apneas associated with respiratory effort throughout the entire period of the event. Mixed apneas were defined as apneas with absent respiratory effort during one portion of the event and presence of respiratory effort in another portion of the event. Hypopneas were identified if there was a ≥ 30% drop in airflow for the duration of at least two breaths associated with a ≥ 3% oxygen desaturation.

### SG procedure technique

The SG procedure was performed using five laparoscopic ports in a standard split-leg French position. Devascularization of the greater curvature of the stomach was done starting from 4 to 6 cm from the pylorus and up to the angle of His before a 36-Fr calibrating bougie was passed through the stomach to the duodenum. The sleeve was then performed with a linear laparoscopic stapler. The staple lines are sequentially fired along the bougie toward the angle of His and divide the fundus at a distance of 0.5 to 2 cm lateral to the esophagus. Finally, the bougie was pulled proximally and an assessment of leak was done by injection of 100 ml of methylene blue. No intra-abdominal drains were placed. If a hiatal hernia was observed intra-operatively, it was repaired during the SG procedure.

### Post-operative follow-up

Patients were typically discharged after 1–3 days post-operatively if vitally stable and had no signs of complications. Afterwards, visits were scheduled for the patients in the clinic at the following intervals: 2 weeks, 3 months, 6 months, 1 year, and 18 months post-operatively.

Patients were also scheduled for visits with dieticians which provided them with nutritional education post-op. For the first 2 days, patients were only allowed sips of water. Afterwards, if tolerated, a diet of clear low-fat, low carbohydrate fluid with protein supplements were initiated for the following 2 weeks, along with an exercise regimen compromising of light walking. From the period of 2 weeks – first month, the patients were given a diet of puréed food consisting of low-fat, high-protein contents. Following that period, a normal reducing diet of small frequent meals was advised, focusing on proteins, with physical activity as tolerated. Multivitamin supplements were prescribed throughout, along with calcium and vitamin D tablets. No gallstone prevention medications were prescribed post-op as there are no clear indications to do so in the bariatric world. If patients presented with severe GERD, an EGD would have been scheduled to evaluate for reflux esophagitis and/or Barret’s esophagus.

### Comorbidity resolution definitions

Hypertension resolution was defined as a blood pressure less than 140/80 mmHg systolic/diastolic, with the lack of usage of antihypertensive medications.

Diabetes was defined as a fasting blood glucose (FBG) level of > 7.0 mmol/L and an HbA1c of more than 6.5%. Concurrently, diabetes resolution was defined as an FBG of < 7.0 mmol/L and HbA1c < 6.5% in the absence of hypoglycemic medications being taken by the patients.

### Statistical analysis

Statistical analysis of the data was carried out using SPSS software version 22. The significance of the difference between the two values was analyzed using a two-tailed unpaired Student’s t test. Significant levels were assessed at p-value < 0.05. Percent Excess Weight Loss (%EWL) was calculated using an ideal body weight equivalent to a BMI of 25 kg/m^2^.

## Results

The total number of patients that underwent laparoscopic sleeve gastrectomy in their adolescent years amounted to 164. The patients age at surgery ranged from 12 to 21 years old (mean age 19). The majority (71%) were female (Table 1).

**Table 1:** Patient Demographics Pre-Operatively

Demographics	Number (SD)/Percentage
Total Number of Patients	164
Age at Surgery (Years)	18.85 (1.92)
Gender Male Female	47 (28.85%) 118 (71.15%)
Weight (Kg)	128.57 (25.59)
BMI (Kg/m^2^)	47.78 (8.9)
Length of Stay (days)	3.62 (4.45)

Mean weight at surgery was 128.6 kg corresponding to a BMI of 47.8 Kg/m^2^ (range 33–105). Fifty two patients (32%) were classified in the BMI more than 50 kg/m^2^ category (BMI > 50), of which eleven (6.7%) presented with a BMI > 60 kg/m^2^ (Table 2).

**Table 2:** Weight Change Parameters with Time. Change in Weight, BMI, %EWL and %TWL over the 13 year follow-up period

Time post-op	Weight (Kg) (SD)	BMI (Kg/m2) (SD)	%EWL	%TWL
2 weeks (n = 114)	120.3 (25.4)	45.3 (9.2)	14.66%	6.79%
3 months (n = 95)	105.7 (24.7)	39.9 (8.8)	39.86%	17.99%
6 months (n = 77)	95.6 (22.3)	36.1 (8.6)	57.82%	26.67%
1 year (n = 52)	82.7 (17.7)	31.3 (7.0)	77.03%	34.51%
18 months (n = 16)	76.0 (10.9)	29.7 (5.3)	82.66%	41.00%
7 years (n = 14)	83.9 (20.3)	31.8 (8.6)	74.58%	36.45%
8 years (n = 24)	90.0 (20.3)	33.3 (7.4)	60.23%	28.41%
9 years (n = 34)	89.7 (25.7)	33.8 (9.4)	65.96%	30.30%
10 years (n = 32)	85.9 (19.6)	31.8 (6.9)	69.94%	32.36%
11 years (n = 14)	87.0 (29.8)	32.9 (10.2)	74.23%	34.86%
12 years (n = 6)	88.3 (23.9)	32.6 (9.5)	70.11%	33.39%
13 years (n = 2)	81.0 (12.7	32.0 (8.4)	78.61%	34.23%

**Fig. 1:**
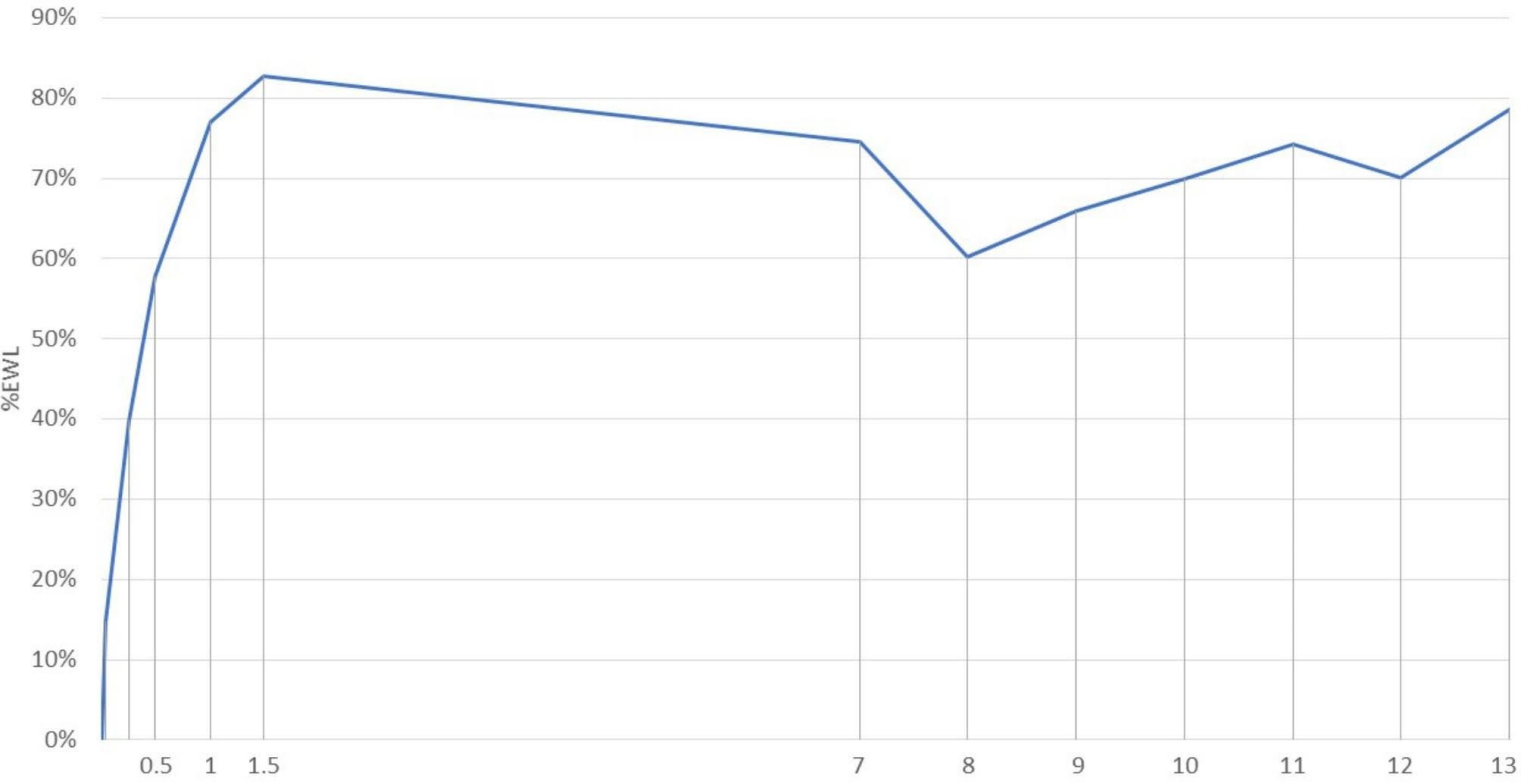
Percent excess weight loss with time of patients over the 13 year follow up

The patients in our study population were followed initially at 2 weeks, 3 months, 6 months, 12 months and 18 months. At the time of our analysis, all patients had reached 7 year follow up. The majority (54%) of patients were followed up at 9 or 10 years post-SG. Weight, BMI, and %EWL were measured at each follow up interval. After two weeks post SG, most patients reached an average weight of 120 Kg, corresponding to a BMI of 45 Kg/m^2^ and EWL of 14.66%. The highest weight loss was recorded at 18 months post-op, corresponding to an average weight of 76 Kg, BMI of 30 Kg/m^2^, EWL of 82.66% and % Total Weight Loss (TWL) of 41%. Long term follow up displayed rather similar results between 7 and 13 years, with values fluctuating between 81 and 90 Kg, BMI between 31.8 and 33.8 Kg/m^2^, and EWL between 60.23 and 78.61%. (Table 2). Figure 1 illustrates the trend in EWL over the years, depicting a steep increase in EWL in the first 18 months, followed by a slight drop afterwards, while stabilizing with minimal variation after several years. Insufficient weight loss is defined as excess weight loss percentage (EWL%) of < 50% 18 months post-bariatric surgery [12]. At 1 year, 8/52 patients (15%) exhibited insufficient weight loss, while at 18 months, 1/16 (6.25%) had insufficient weight loss.

Weight regain is defined as progressive weight regain that occurs after achievement of an initial successful weight loss (defined as EWL > 50%) [12]. 5 patients out of the 164 that we examined exhibited weight regain throughout the follow-up period of 13 years. 1 patient began exhibiting weight regain at 8 years post-op, 3 at 10 years post-op and 1 at 12 years post-op.

Evaluation of major comorbidities at preoperative screening revealed 8 patients (5%) diagnosed with obstructive sleep apnea (OSA), 2 patients (1.2%) diagnosed with hypertension (HTN) and 1 (0.6%) patient diagnosed with type 2 diabetes mellitus (DMII) (Table 3). Long term follow-up revealed the resolution of OSA in 75% of these patients, and DMII in the one patient that was diagnosed with it pre-operatively.

**Table 3:** Morbidity and Co-morbidity Resolution

Comorbidities	Number (n)/Percentage (%)
**OSA**	
Pre-op Resolved	8 (4.8%) 6 (75%)
**HTN**	
Pre-op Resolved	2 (1.2%) 0 (0%)
**DM**	
Pre-op Type 1 Resolved	1 (0.6%) - 1 (100%)
**Bleed**	1 (0.6%)
**Leak**	2 (1.2%)
**GERD** **Time until development of GERD (yrs)**	21 (12.7%) 5.69 (4.35)
**Gall Bladder Stones (GBS)** **Time until development of GBS (yrs)**	20 (12.1%) 4.37 (3.59)

Mean hospital stay post-operatively was 3.6 days. No morbidity or mortality was recorded in the immediate post-operative period. Over long-term follow-up however, 1 patient was treated for a bleed, 2 patients were treated for a leak, 21 patients developed gastro-esophageal reflux disease (GERD) 5.7 years post-operatively, and 20 patients were treated for gall bladder stones with a laparoscopic cholecystectomy at 4.4 yeas post-op. No mortality was recorded in our cohort.

## Discussion

Obesity in the younger age groups is becoming an ever-growing problem that manifests its outcomes through adulthood. Laparoscopic sleeve gastrectomy is a bariatric procedure that has proven to be effective for the long-term management of obesity in adults [13–16]. However, long term data on adolescents is currently lacking. One of the main reasons behind this finding is because bariatric procedures, until recently, have been limited to adults [17]. Therefore, we chose to investigate the long-term outcomes of this procedure in adolescents. Our paper was able to prove sustained weight loss over a 10 year follow up period with good outcomes in regards to comorbidity resolution. Furthermore, there were minimal negative outcomes encountered in our cohort over the ten year period, with 12% of our population developing GERD and/or gall bladder stones 5 years post-operatively.

Worldwide in the year 2016, obesity amongst children was estimated to be present in around 60 million of the population [18]. Further, according to the World Health Organization, the prevalence of obesity and overweight in that age-group increased from 17 to 21% between the years of 2007 and 2019 respectively [19]. In Kuwait alone obesity was recorded at a record high rate of 33% amongst adolescent age groups in 2019 [20]. Therefore, the management of this disease has become imperative to prevent lifelong sequalae in this population. Recent reports have been able to prove that laparoscopic bariatric procedures in the adolescents is not only safe, but demonstrate incredibly successful results in terms of weight loss and comorbidity resolution [21–23].

It is important to bear in mind that outcomes of laparoscopic sleeve gastrectomy are influenced by many factors such as adherence to the post-operative regimen, lifestyle, diet, and follow up appointments. Nonetheless, we were able to show a decline in post-operative BMI from 47.8 kg/m^2^ to 31.3 kg/m^2^ 12 months post-SG. This was in line with results obtained in older patients undergoing SG in our hospital [24]. The study also proved that the vast majority of our population were able to maintain their BMI even after a 10 + year follow-up period. These weight loss results were further paralleled regionally in studies from Qatar as well as the United States [25, 26]. In a similar pattern the %EWL followed the BMI changes as such that after 12 months from the procedure, approximately 77.03% EWL was achieved, which was maintained 13 years post-operatively. This provides insight on the long-term effectiveness of bariatric surgeries such as the one conducted in our research [4].

SG is a helpful tool that aids in weight loss which can help with the metabolic manifestations associated with obesity. It has been proven to lower the undesirable complications such as hypertension, diabetes, and hypercholesterolemia [4, 25]. However, these outcomes were not fully assessed in our study mainly because the majority of our study participants did not suffer from the complications to begin with. However, the 2 patients that presented with hypertension pre-operatively did not demonstrate resolution of their hypertension 10 years post-op. A possible explanation for the lack of complete hypertension resolution might be related to other factors, such as low vitamin D levels, which were not assessed in our patients. A previous study observing the effect of hypovitaminosis D on the resolution of hypertension among patients undergoing Roux-en-Y Gastric Bypass (RYGB) interestingly found that vitamin D reduction was significantly associated with a lack of resolution of HTN compared to those with adequate levels (42 vs. 61%; p = .008) [27].

Since bariatric surgery such as SG is known to cause rapid weight loss and hence, increase the development of gall stones [28], it was witnessed in our study that approximately 20 of the participants (12.1%) developed gall bladder stones at around 4 years post-op. This has been seen to be a common finding in post-bariatric surgery patients as shown by previous studies [29–31]. Further, females have been proven to be at a greater risk of developing cholelithiasis, with a female to male ratio of 2.1:1 [32], and given that the majority of our patients, as well as patients undergoing bariatric procedures, are female (71.1% of our cohort), this high rate of gallstone formation is not surprising. When it came to looking at other morbidities encountered post-operatively, 21 (12.7%) patients developed GERD approximately 5 years later, while a leak was encountered in 1.2% of the patients. This rate is lower than that of the average leak rate encountered post primary SG which is approximately 2.2% [33]. Further, the development and presence of malnutrition in adolescents is a worrying consequence that may present following restrictive bariatric procedures. Xanthakos et al. [34] was able to demonstrate that after RYGB, but not SG, serum concentrations of vitamin B12 significantly decreased whereas serum levels of transferrin and parathyroid hormone increased, while ferritin levels decreased significantly after both procedures 5 years post-op. However, no other studies were able to demonstrate a significant affect of sleeve gastrectomy’s on nutritional or vitamin status of adolescent patients mid- to long-term. Further, previous studies conducted by Alqahtani et al.[35, 36] were able to show that all patients at the different age groups (ranging from 5 years old to 21 years old) experienced normal growth velocity throughout the study period of 10 years, representing no significant change in growth velocity. The development of gastroesophageal reflux disease post sleeve gastrectomy is a known possible sequelae that could arise and be of worry to control, especially if it develops at a younger age and carries through adulthood. A recent systematic review by Pavone et al. [37] examining this topic, however, was able to come to the conclusion that satisfactory control of postoperative reflux can be achieved in most patients, while low rates of de novo GERD develop, provided a tubular cuff is created when operating on an obese patient that presents with GERD pre-operatively, as recently stated in the 5th International Consensus Conference on sleeve gastrectomy [38]. Assuredly, many studies have been able to demonstrate improved incidence of GERD post-sleeve, especially in patients that suffered from it pre-op [39, 40] by repairing hiatal defects systematically and paying careful attention to surgical technique, avoiding torsion or narrowing of the sleeve. One study looked at the incidence of de-novo GERD in pediatric and adolescent patients that underwent sleeve gastrectomy [41] and was able to conclude that even though the incidence is small (5.65–16.67%), it deserves attention.

Adolescent’s have been shown to have a higher risk of follow-up interruption than adults, mainly due to their more mobile lifestyle at that age (university, employment or personal life changes). This phenomenon can be witnessed in our study by the fact that 87 (53%), 112 (68%) and 132 (80%) patients that were eligible for evaluation at 6 months, 1 year and 10 years after SG were lost to follow-up, respectively. Therefore, emphasis on adherence to the post-operative medical treatment and team is an essential key for the successful treatment post-bariatric surgery in pediatric and adolescent patients [42].

Our study presents with several limitations that should be highlighted. Firstly, our study is one that it is a retrospective case series without the presence of a control group. However, it is one of the fewest published in this region with a long follow-up period targeting the adolescent age group in a geographic location which is heavily burdened by obesity. Second, even though this is one of the largest single-series studies conducted on this population of bariatric patients, the total number of patients is still small. Thirdly, the number of patients followed up decreased significantly over the follow-up period, which is an issue seen in adolescent patients, and therefore decreases the strength of the long-term evaluation post-SG.

## Conclusion

Obesity is an ever-growing dilemma which can be rooted early in life from the beginning of childhood. It is of ample importance to tackle obesity before complications ensue later on in life. In the many occasions when behavioral interventions fail to correct an individual’s weight, bariatric surgery was found to be the life-saving intervention. The same could be paralleled onto adolescents in which SG was found to be a safe and effective weight loss tool by our study, with sustained long-term weight loss, as well as resolution of early comorbidities.

## Data Availability

The datasets used and/or analysed during the current study available from the corresponding author on reasonable request.
